# The Inverse Association between the Frequency of Forest Walking (Shinrin-yoku) and the Prevalence of Insomnia Symptoms in the General Japanese Population: A Japan Multi-Institutional Collaborative Cohort Daiko Study

**DOI:** 10.3390/ijerph21030350

**Published:** 2024-03-15

**Authors:** Emi Morita, Hiroshi Kadotani, Naoto Yamada, Tae Sasakabe, Sayo Kawai, Mariko Naito, Takashi Tamura, Kenji Wakai

**Affiliations:** 1 International Institute for Integrative Sleep Medicine (WPI-IIIS), University of Tsukuba, 1-1-1 Tennodai, Tsukuba 305-8575, Japan; 2 Forestry and Forest Products Research Institute, Forest Research and Management Organization, 1 Matsunosato, Tsukuba 305-8687, Japan; 3 Department of Psychiatry, Shiga University of Medical Science, Seta Tsukinowa-cho, Otsu 520-2192, Japan; kadotani@belle.shiga-med.ac.jp (H.K.); n-yamada@anzu.or.jp (N.Y.); 4 Kanbayashi Memorial Hospital, 89-1 Orikuchinishi, Okucho, Ichinomiya 491-0201, Japan; 5 Department of Preventive Medicine, Nagoya University Graduate School of Medicine, 65 Tsurumai-Cho, Nagoya 466-8550, Japan; tsasa@aichi-med-u.ac.jp (T.S.); ttamura@med.nagoya-u.ac.jp (T.T.); wakai@med.nagoya-u.ac.jp (K.W.); 6 Department of Public Health, Aichi Medical University School of Medicine, Nagakute 480-1195, Japan; 7 Department of Oral Epidemiology, Graduate School of Biomedical and Health Sciences, Hiroshima University, 1-2-3 Kasumi, Minami-ku, Hiroshima 734-8553, Japan; naitom@hiroshima-u.ac.jp

**Keywords:** sleep, forest bathing, Shinrin-yoku, epidemiology, cross-sectional study

## Abstract

Since a single forest walk (Shinrin-yoku or forest bathing) session is reported to improve sleep temporarily, occasional forest walks may have a positive effect on daily sleep. Therefore, this study aimed to examine whether more frequent forest walking is associated with better daily sleep conditions. Data from the second survey of the Japan Multi-Institutional Collaborative Cohort (J-MICC) Daiko Study conducted among residents of Nagoya City, Japan, were used. The study design was a cross-sectional study. In total, 2044 participants (529 men and 1515 women; age, mean ± standard deviation: 58.8 ± 9.9 years) were included in the analysis. Frequent forest walks were associated with a low percentage of insomnia symptoms (Insomnia Severity Index ≥10) in women, but not in men. The adjusted odds ratio for the group that rarely took forest walks with reference to the group that engaged in the activity once a month or more often was 2.04 (95% confidence interval: 1.29–3.23) in women. Forest walk frequency was not significantly associated with sleep duration or sleep efficiency as measured by actigraphy in either men or women. In conclusion, the results suggested that increasing the frequency of forest walks or Shinrin-yoku may be effective in preventing insomnia in women.

## 1. Introduction

It is empirically known that some natural environments contribute to the promotion and maintenance of health. In several European countries, health resort medicine is a form of healthcare provision, and most health resorts are located in natural environments [1]. Epidemiological studies have also reported the health advantages of green spaces. A study on the relationship between health inequalities and green space involving 40,000,000 participants in the UK showed that health inequalities related to income deprivation in terms of all-cause mortality and mortality from circulatory diseases were low in the greenest areas [2].

In Japan, forest walking or Shinrin-yoku (walking and/or staying in forested areas) is a well-known health promotion activity. This is because the total forest area in the world is 31% of the total land area; however, in Japan, forests cover 67% of the land area, making forests the most typical natural environment [3]. Shinrin-yoku was first proposed in the 1980s in Japan and is considered to promote both physical and mental health via the inhalation of substances released from trees and via exercise and/or other healing factors associated with the forest environment [4]. Shinrin-yoku may also be translated as “forest bathing”. It is a health promotion method in which the intensity of the exercise does not matter, and no special skills are required, as the focus is on exposure to the forest environment. Therefore, individuals who take a walk in the forest or stay in a forest environment are performing Shinrin-yoku. According to a public opinion poll conducted in 2011 by the Cabinet Office of the Government of Japan, 37.2% of the respondents had participated in Shinrin-yoku within the preceding year to refresh their mental and physical conditions [5]. In recent years, Shinrin-yoku, or forest therapy, has also been adopted and studied in countries outside Japan [6,7]. Furthermore, theories on the effects of Shinrin-yoku are also being explored [7]. In many cases, forest therapy methods are accompanied by specific programs, such as the programs in health resort medicine in Germany.

Dozens of studies have been conducted on the effects of Shinrin-yoku, forest walking, or comprehensive forest therapy programs that may involve overnight stays [6,7,8,9,10,11]. Most of the available studies to date are interventional and have examined the acute effects of engaging in a single session of forest walking, Shinrin-yoku, or forest therapy programs [6,7,8,9,10,11]. A meta-analysis of blood pressure reported that forest environments contribute significantly to lower blood pressure compared with non-forest environments [8]. Psychological benefits, particularly reduced anxiety [6,9], lower levels of stress-related hormones such as cortisol [10], and beneficial changes in immunological and inflammatory parameters [6], have also been reported. Thus, a single Shinrin-yoku session has been reported to have acute positive effects.

However, studies of a single session of forest walking cannot assess the effect of forest walks on health because the acute temporary effects eventually disappear. Since maintaining a healthy activity has a positive impact on health in general, occasional forest walking may positively influence health. Epidemiological studies with populations are needed to assess daily health effects. However, epidemiological studies examining the association between Shinrin-yoku and daily health are limited [12,13].

Regarding sleep, it was reported that in those dissatisfied with their sleep, sleep duration as measured by actigraphy was extended, and subjective sleep quality improved on the night after a single 2 h forest walking session [14]. Middle-aged men who took two 2 h Shinrin-yoku sessions during the 3-day trip also reported improved self-rated sleepiness on rising and refreshing the next morning [15].

Good sleep is essential for health promotion and disease prevention. Sleep disturbance, however, is a major health problem in Japan. Among the Organization for Economic Co-operation and Development (OECD) countries, Japan has reported the shortest sleep duration in its population [16]. In a national survey from 2007, the prevalence of insomnia symptoms, which include difficulty falling asleep, difficulty maintaining sleep, and early morning awakening, was 38.6% in men and 34.6% in women in the general population [17]. Since sleep disturbance has been reported as a risk factor for depression and suicide [18,19,20,21], it is important to improve sleep in the general population rather than merely focusing on patients with insomnia. Therefore, concrete and practical methods to improve sleep that are applicable in daily life are necessary.

Considering that forest walking has a temporary, acute effect on sleep improvement, forest walks at certain intervals could improve daily sleep and contribute to the prevention of sleep-related diseases. However, to the best of our knowledge, very few epidemiological studies have examined whether individuals who take forest walks have good daily sleep.

Therefore, this study aimed to evaluate whether the frequency of forest walking is associated with daily sleep conditions, including insomnia symptoms, in the general Japanese population.

## 2. Materials and Methods

Data from the sleep assessment section of the second survey of the Japan Multi-Institutional Collaborative Cohort (J-MICC) Daiko Study were used. The J-MICC study is a long-term cohort study that investigated interactions between genetic factors, lifestyle, and lifestyle-related diseases (especially cancer) [22]. The J-MICC Daiko study was conducted by a research team at Nagoya University, one of the teams that participated in the multicenter J-MICC studies.

**Figure 1:**
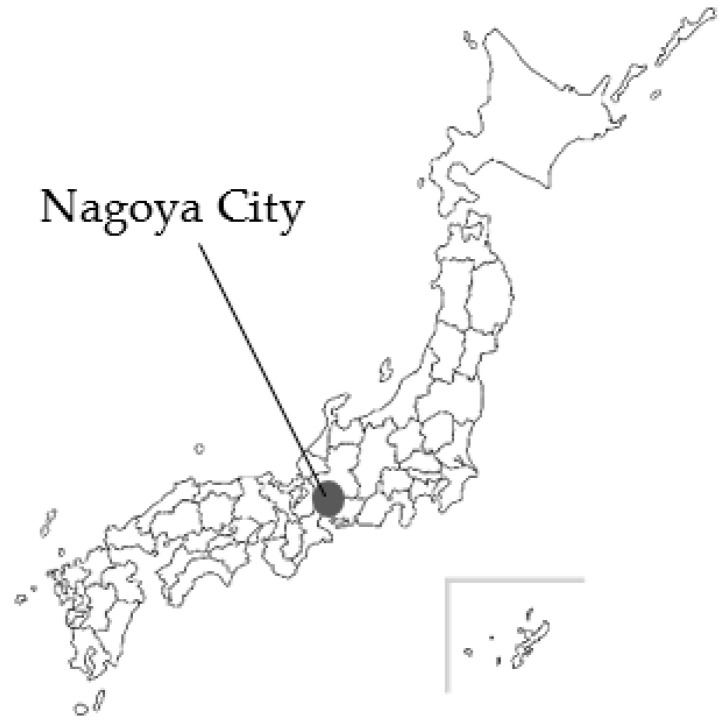
Location of Nagoya City in Japan.

The baseline survey of the J-MICC Daiko study was conducted between June 2008 and May 2010. Details of the study protocol, including participant recruitment, data collection, measurement methods, and participant characteristics, have been previously reported [23]. The recruitment criteria for participants were that they were aged 35–69 years at the time of the baseline survey and resided in Nagoya City, a large urban center located in the central part of the main island of Japan (Figure 1). Participants were recruited mainly via a citywide mailbox distribution of leaflets and personal communications between prospective participants [23].

The second survey was conducted in 2014, approximately five years after the baseline survey. Participants from the baseline study were invited to participate in the second survey. The survey included a questionnaire, medical examination, sleep measurements, and blood sample collection.

### 2.1. Participants

The participants of the second survey of the J-MICC Daiko study who had consented to the sleep assessment were included in the present study. Those who did not meet the inclusion criteria (due to incomplete actigraphy data or missing data for frequency of forest walking or lifestyle) were excluded. Shift workers were also excluded. Sleep measurement with actigraphy was requested for 1 week, and those who performed the measurement for at least three nights were included in the study. Finally, 2044 participants (529 men and 1515 women; age, mean ± standard deviation [SD]: 58.8 ± 9.9 years; range, 39–75) were included in the analysis.

### 2.2. Sleep Measurements

The participants were required to wear an actigraphy device on the waist (MTI-210, ACOS Co., Ltd., Nagano, Japan) for 24 h a day (except during bath time) during an entire week, to estimate actual sleep time and sleep efficiency [24,25]. Measurements obtained using a waist-worn actigraphy device with a built-in sensor equivalent to the MTI-210 have been reported to correlate well with polysomnography results [24]. The device has shown a high degree of correlation when worn on the body and on the wrist [25]. The participants were also instructed to record their bed time and get-up time in a sleep diary. The average number of nights was 6.9 ± 0.6 nights (3 to 10 nights); 85.9% of the participants performed the 7-night measurement as requested.

When the actigraphy data were analyzed, bed time and get-up time were determined based on sleep diaries. The actigraphy data were analyzed using the Sleep Sign Act software v. 1 (Kissei Comtec Company Inc., Matsumoto City, Japan) [25]. Sleep duration per day was defined as the sum of all recorded sleep durations, including naps, within a 24 h period. The calculation of sleep efficiency included only the longest sleep period within 24 h (from noon to noon of the next day) and did not include naps. The average value during the measurement period was used to analyze sleep duration and sleep efficiency.

### 2.3. Questionnaire

The participants were requested to complete a self-administered questionnaire that recorded the following data: frequency of forest walking (“How frequently do you perform forest walking, including hiking, nature observation, mountain-walking, working or camping in a forested area, excluding visits to city parks?”; 6 categories, 1: once a week or more, 2: two or three times a month, 3: once a month, 4: several times a year, 5: once a year, and 6: rarely); lifestyle, for example, smoking status and alcohol consumption; and leisure-time activities (intensity, frequency, and duration of exercise).

Insomnia symptoms and self-rated sleep quality were evaluated by the Insomnia Severity Index (ISI) and Pittsburg Sleep Quality Index (PSQI), respectively. The ISI comprises seven items, and the higher the total score (range: 0–28), the worse the condition [26,27]. A cut-off score of 10 has been reported to be optimal for detecting insomnia in a community [28]. The PSQI assesses sleep quality and disturbances over 1 month and is frequently used in sleep science. PSQI is assessed by a global score of 18 items consisting of the following seven components: sleep quality, sleep latency, sleep duration, habitual sleep efficiency, sleep disturbance, use of sleeping medication, and daytime dysfunction. The higher the global score (range: 0–21), the worse the condition [29,30]. Chronotype was evaluated using the Morningness–Eveningness Questionnaire (MEQ), which consists of 19 items and classifies the users into one of the following chronotypes according to their scores: definite evening (score: 16–30), moderate evening (score: 31–41), intermediate (score: 42–58), moderate morning (score: 59–69), and definite morning (score: 70–86) [31,32]. The Japanese versions of these three questionnaires were used [27,30,32].

### 2.4. Statistical Analysis

The six categories used to classify the frequency of forest walking were converted into four categories in this study, with the first three categories (once a week or more, several times a month, and once a month) combined into a single category (“once a month or more”). Body mass index (BMI) was calculated using weight and height measurements and classified into two categories: ≥25.0 and <25.0 kg/m^2^. Habitual nightcap use was defined as drinking alcohol within 2 h before bed at least once a week. Habitual exercise was defined as ≥30 min of leisure time activity at least once a week [13].

PSQI scores >5.5 indicated poor sleep quality [29]. ISI scores ≥10 indicated the presence of insomnia symptoms. Although classification according to the MEQ typically involves five categories, they were converted into three in the present study: “eveningness” (definite evening and moderate evening; score: 16–41), “intermediate” (score: 42–58), and “morningness” (moderate morning and definite morning; score: 59–86).

The percentage difference in the frequency of forest walking according to sex was calculated using the chi-square test. The associations between ordinal variables were analyzed using the Mantel–Haenszel test for trends. The trends in continuous variables by ordinal categories were tested using a linear regression model.

In logistic regression analysis in Model 1, the dependent variable was insomnia symptoms (yes/no) or poor sleep quality (yes/no). In contrast, the independent variables were age (39–49, 50s, 60s, and 70s), BMI (≥25.0/<25.0), smoking status (current smoker/other responses), habitual nightcap use (once a week or more/other responses), habitual exercise (leisure time activity: once a week for at least 30 min or more/other responses), and frequency of forest walking (four categories). In Model 2, the dependent variable was also insomnia symptoms or poor sleep quality, whereas the independent variables were the same as in Model 1 plus chronotype based on the MEQ (three groups: morning type, intermediate type, evening type). The significance level was set at 5%. IBM SPSS Statistics version 23 for Windows (IBM, Armonk, NY, USA) was used for statistical analysis.

## 3. Results

The frequency of forest walking was 9.9% (*n* = 203) for “once a month or more”, 22.8% (*n* = 466) for “several times a year”, 12.9% (*n* = 264) for “once a year”, and 54.4% (*n* = 1111) for “rarely”. The characteristics of the participants discriminated by their frequency of forest walking are shown in Table 1.

The frequency of forest walking differed significantly by sex, with a higher percentage among men (*p* = 0.001). For both men and women, those with a higher frequency of forest walks were significantly older (*p* = 0.02 for men, *p* < 0.001 for women) and had significantly higher percentages of exercise habits (*p* < 0.001 for men, *p* < 0.001 for women). Chronotypes assessed by MEQ were significantly associated with the frequency of forest walking in women (*p* < 0.001) but not in men (*p* = 0.58). BMI and lifestyle habits such as nightcaps and smoking were not associated with the frequency of forest walking for either men or women.

The mean sleep duration evaluated by actigraphy was 355.0 ± 59.5 min (range: 116.3–573.1). The mean sleep efficiency was 82.0 ± 9.9% (range: 27.1–97.5). Table 2 shows the sleep duration and sleep efficiency of the participants evaluated by actigraphy discriminated according to their frequency of forest walking. There was no significant difference in either sleep duration or sleep efficiency between the frequencies of forest walking for either men or women.

The number of participants with poor self-rated sleep quality (PSQI ≥ 6) was 884 (43.2%), and that of participants with insomnia symptoms (ISI ≥ 10) was 628 (30.7%). No significant differences were observed in the percentage of poor sleep quality (42.0% for men, 43.7% for women, *p* = 0.49) or the presence of insomnia symptoms (men 29.9% for men, 31.0% for women, *p* = 0.62) between men and women.

The proportions of participants with poor sleep quality and insomnia symptoms discriminated by frequency of forest walks are shown in Table 3.

For both men and women, the percentage of poor sleep quality was inversely associated with the frequency of forest walking (men; trend *p* = 0.009, women; trend *p* = 0.04). There was no statistically significant association between the proportion of participants with insomnia symptoms and the frequency of forest walks in men (trend *p* = 0.82). However, a statistically significant inverse association was found among women, with a lower percentage of those in the more frequent forest walking group reporting insomnia symptoms (trend *p* = 0.002).

Table 4 shows the results of the logistic regression analyses. For women, the significant difference in poor sleep quality (PQSI > 5.5) according to the frequency of forest walks disappeared after adjusting for potentially relevant factors. For men, differences in the frequency of forest walking reached statistical significance in poor sleep quality, but the adjusted odds ratio (aOR) for each category when “once a month or more” was used as a reference were not significantly different. Regarding the percentage of participants with insomnia symptoms (ISI ≥ 10), there was no significant difference in the frequency of forest walks among men, even after adjusting for potentially relevant factors, while a significant difference in the frequency of forest walks was found in women, even after adjusting for potentially relevant factors. The aOR when “once a month or more” was considered as a reference was 1.66 (95% confidence interval [CI]: 1.01–2.72) for “several times a year to more than once a month”, 1.86 (95%CI: 1.09–3.16) for “once a year”, and 2.04 (95%CI: 1.29–3.23) for “rarely” in the fully adjusted Model 2.

In the logistic regression analysis, exercise habits were not significantly associated with poor sleep quality or insomnia symptoms. Regarding the presence of insomnia symptoms, the aORs for exercise habits were 0.94 (95% CI: 0.61–1.44) for men and 0.80 (95% CI: 0.63–1.01) for women in Model 1, and 0.94 (95% CI: 0.61–1.44) for men and 0.81 (95% CI: 0.64–1.03) for women in Model 2. The aORs were less than 1, but no significant association was found in either case. Regarding poor sleep quality, the aORs for exercise habits were 0.82 (95% CI: 0.55–1.22) for men and 0.95 (95% CI: 0.76–1.19) for women in Model 1, and 0.84 (95% CI: 0.56–1.26) for men and 0.97 (95% CI: 0.78–1.21) for women in Model 2, with no significant association in either case.

## 4. Discussion

This study examined whether the frequency of forest walking is related to good daily sleep in the general population. The results showed that among women, a higher frequency of forest walking was associated with a lower percentage of insomnia symptoms. This suggests that increasing the frequency of forest walking may be effective in preventing insomnia in women. In this study, even the highest frequency of forest walks was “once a month or more”, which is not as frequent as the regular practice of general exercise. Even a frequency of “once a month” or “several times a year” was shown to be potentially beneficial for the prevention of insomnia.

A previous study reported that a single forest walking session can improve sleep acutely and temporarily [14]. The present study showed that forest walks can potentially prevent insomnia and other diseases caused by insomnia in women beyond a temporary, acute effect. For men, logistic regression analysis showed an association between forest walk frequency and sleep quality. Nevertheless, the adjusted odds ratios for each category of forest walk frequency did not reach a statistical significance. This may be because the number of male participants was not large enough. The number of men in this study was only approximately one-third of the number of women. If the number of men had been higher, the odds ratios for each category of forest walk frequency might have reached statistical significance.

Regarding objective sleep assessment, the frequency of forest walks was not associated with objectively measured sleep duration or sleep efficiency. However, these results are not contradictory because insomnia is a subjective disease. A patient’s complaint of insomnia is a diagnostic criterion, while objective sleep duration is not. Objective measurements, such as polysomnography and the Multiple Sleep Latency Test, are usually not helpful in the establishment of an insomnia disorder diagnosis [33]. However, a study that examined the acute effects of a single session of forest walking reported increased objectively measured sleep duration and improved subjective sleep quality on the night after forest walking [14]. There was no difference in daily sleep duration based on forest walking frequency in the present study, which is inconsistent with those of the study mentioned above. The first reason for this discrepancy could be due to differences in the target population. The acute effect study involved people who were dissatisfied with their sleep, so the forest walking intervention may have been more likely to increase sleep duration. The present study, on the other hand, involved the general population. The second reason is that the highest frequency of forest walking in our study was “more than once a month”, implying that it was not a daily activity. Even if sleep duration increased on the night of the forest walk, social schedules took precedence in daily life, and daily sleep duration may not have been longer. The population of Japan reportedly has the shortest sleep duration compared to those from other OECD countries, which is considered to be due to the constraints of social life.

Regarding subjective sleep assessment, one or two forest walk sessions have been reported to acutely improve subjective sleep state [14,15]. In this epidemiological study, the group with a higher frequency of forest walks also had a lower percentage of participants with subjective insomnia symptoms, indicating that both the acute effect study and this epidemiological study suggest that forest walks or Shinrin-yoku may improve subjective sleep status. Two possible reasons why forest walking or forest bathing may positively influence subjective sleep are exercise and psychological refreshment. Regarding exercise, even the highest frequency of forest walking, “once a month or more”, is too infrequent to be considered an exercise habit. Therefore, it is unlikely that exercise via forest walking has a direct effect on daily subjective sleep. Additionally, logistic regression analyses showed that habitual exercise, independent of the frequency of forest walks, was not associated with insomnia symptoms. Thus, insomnia symptoms were associated with the frequency of forest walks and not with exercise habits, suggesting that forest environments rather than exercise may be beneficial for preventing or alleviating insomnia symptoms. Hence, it is conceivable that regular psychological refreshments in a forest environment could have a positive impact on subjective sleep.

These findings suggest that the forest environment may play a positive role in improving insomnia in women. Although not forests, epidemiological studies of green spaces in several countries have reported an association between exposure to green space environments and sleep [34,35,36,37,38,39,40]. These studies show that contact with green environments may positively affect sleep.

In many parts of Japan, forests are accessible within an hour, as is the case in Nagoya, the large urban center that was the region of this study. The Aichi Prefecture, of which Nagoya City is a part, has a forest coverage of 42% of the land area, while the national average is 68.4% of the total land area [41,42]. Thus, it is feasible to take a forest walk at least once a month to improve health. Shinrin-yoku is increasingly being practiced outside Japan [6] and can be undertaken anywhere in forests with suitable climates. Since forest walking does not require specific techniques and is, therefore, a widely accessible activity, it may be a practical method to improve sleep that is easily implemented.

This study addressed only the frequency of forest walks and did not investigate the duration or the distance covered per walk. Future epidemiological studies may benefit from considering these variables as well. However, the finding of a significant association for frequency alone is simple and easy to incorporate into practice. It should also be noted that in a study examining the acute psychological effects of walking in a forest environment, neither distance nor duration were associated with psychological effects [43].

There were several limitations to this study. The first was related to the study design. We could not clarify causal relationships because this was a cross-sectional study. People who have a lifestyle that allows for frequent forest walks may have more time to spare in their daily lives, which may result in less insomnia. Cohort studies are needed to examine causal relationships. Moreover, as this was an epidemiological study, it was not possible to elucidate the mechanism by which forest walking contributes to the prevention of insomnia symptoms. Second, regarding the insomnia symptoms, the results differed between men and women, but the cause of the difference could not be determined. There were fewer men than women in the present study, and therefore it is possible that there were not enough men to consider. Alternatively, unknown factors not included in the adjustment factors in this study may have been sex-related, thereby causing sex differences in the results. Third, epidemiological studies cannot examine details, such as what spaces and courses are best for sleep health. Such details should be examined in intervention studies. Further studies should be conducted in both, considering the strengths and limitations of epidemiologic and intervention studies. Fourth, as discussed above, exercise habits, one of the independent variables in the logistic regression analysis, is an important factor. However, exercise habits were not defined using objective data. Instead, as common in epidemiological studies, it was defined only by the frequency and time per session reported by the participants via the questionnaire.

Nevertheless, the strength of this study is that the association between forest walking or Shinrin-yoku and daily sleep conditions was verified via an epidemiological study using data from a large-scale survey with 2044 participants.

The future prospects are as follows: Epidemiological studies sometimes show inconsistent results in different populations, even in those with similar backgrounds. Therefore, the results reported here need to be validated in other populations, and additional data needs to be collected. Although the participants in this study belonged to a general population of local residents, each study needs to be validated with a population appropriate for the purpose of the study, e.g., in the general population of university students, working people, older adults, or in a group of patients with sleep-related diseases. Furthermore, the natural environment and accessibility to forests vary from region to region, and especially from country to country, and should therefore be evaluated in different regions in the future. Epidemiological studies on the benefits of visiting forest environments are still limited. Hypertension among local residents and stress-coping skills among workers have been reported [12,13], and this study examined sleep among local residents. In the future, additional outcomes such as lifestyle-related diseases, cancer, mortality, and well-being should also be examined.

## 5. Conclusions

A single forest walk (Shinrin-yoku or forest bathing) session has been reported to improve sleep temporarily. However, studies of a single session of forest walks cannot assess the effect of forest walks on chronic sleep states. Since maintaining a healthy activity positively influences health in general, occasional forest walking may have a positive impact on daily sleep. Epidemiological studies are needed to assess this possibility. Hence, this study examined data from an epidemiological cross-sectional study of 2044 participants to examine whether more frequent forest walking is associated with better daily sleep conditions.

The results showed that among women, a higher frequency of forest walking was associated with a lower percentage of insomnia symptoms. The adjusted odds ratio for the group that rarely took forest walks with reference to the group that engaged in the activity once a month or more often was 2.04 (95% confidence interval: 1.29–3.23). This suggests that increasing the frequency of forest walking, or Shinrin-yoku, may be effective for the prevention of insomnia in women. These findings show that forest walking not only has a temporary effect on sleep but also that more frequent forest walking may contribute to daily sleep conditions, an important component of health promotion.

Since epidemiological studies may report inconsistent results in different populations, further verification in other populations is required to confirm these findings. In addition, epidemiological studies should be conducted on other outcomes, such as lifestyle-related diseases and well-being. Cohort studies in which causal relationships can be identified are also desirable in the future.

## Figures and Tables

**Table 1:** Characteristics of participants by frequency of forest walking.

Frequency of Forest Walking	Total		Once a Month or More		Several Times a Year		Once a Year		Rarely		*p* Value
	*n*	(%)	*n*	(%)	*n*	(%)	*n*	(%)	*n*	(%)	
Total	2044	(100.0)	203	(9.9)	466	(22.8)	264	(12.9)	1111	(54.4)	
Men	529	(100.0)	72	(13.6)	136	(25.7)	60	(11.3)	261	(49.3)	0.001 ^(d)^
Women	1515	(100.0)	131	(8.6)	330	(21.8)	204	(13.5)	850	(56.1)	
Age: Mean ± S.D.											
Men	60.6 ± 9.9		64.3 ± 8.6		60.3 ± 10.7		59.3 ± 10.3		60.1 ± 9.6		0.02 ^(e)^
Women	58.1 ± 9.7		61.8 ± 9.4		58.6 ± 10.1		57.0 ± 9.5		57.6 ± 9.6		<0.001 ^(e)^
BMI ≥ 25.0											
Men	132	(25.1)	13	(18.1)	39	(28.7)	10	(16.7)	70	(26.8)	0.39 ^(f)^
Women	152	(10.0)	10	(7.6)	42	(12.7)	15	(7.4)	85	(10.0)	0.81 ^(f)^
Nightcap ^(a)^											
Men	161	(30.4)	22	(30.6)	37	(27.2)	24	(40.0)	78	(29.9)	0.82 ^(f)^
Women	161	(10.6)	15	(11.5)	37	(11.2)	22	(10.8)	87	(10.2)	0.55 ^(f)^
Current smoker											
Men	62	(11.7)	7	(9.7)	12	(8.8)	8	(13.3)	35	(13.4)	0.18 ^(f)^
Women	45	(3.0)	3	(2.3)	11	(3.3)	3	(1.5)	28	(3.3)	0.63 ^(f)^
Habitual exercise ^(b)^											
Men	349	(66.0)	63	(87.5)	94	(69.1)	37	(61.7)	155	(59.4)	<0.001 ^(f)^
Women	907	(59.9)	107	(81.7)	240	(72.7)	125	(61.3)	435	(51.2)	<0.001 ^(f)^
MEQ ^(c)^											
Men											
Morning type	231	(43.6)	35	(48.6)	52	(38.2)	28	(46.7)	116	(44.4)	0.58 ^(f)^
Intermediate type	284	(53.7)	35	(48.6)	78	(57.4)	30	(50.0)	141	(54.0)	
Evening type	14	(2.6)	2	(2.8)	6	(4.4)	2	(3.3)	4	(1.5)	
Women											
Morning type	620	(40.9)	72	(55.0)	141	(42.7)	83	(40.7)	324	(38.1)	<0.001 ^(f)^
Intermediate type	847	(55.9)	57	(43.5)	185	(56.1)	113	(55.4)	492	(57.9)	
Evening type	48	(3.2)	2	(1.5)	4	(1.7)	8	(3.9)	34	(4.0)	

^(a)^ Once a week or more; ^(b)^ leisure time activity (once a week for 30 min or more; ^(c)^ Morningness–Eveningness Questionnaire; ^(d)^ chi-squared test; ^(e)^ linear regression model; ^(f)^ Mantel–Haenszel test for trend.

**Table 2:** Sleep duration and sleep efficiency measured by actigraphy by frequency of forest walking.

Frequency of Forest Walking	Once a Month or More		Several Times a Year		Once a Year		Rarely		*p* ^(a)^
	*n*	Mean ± SD	*n*	Mean ± SD	*n*	Mean ± SD	*n*	Mean ± SD	
Sleep Duration									
Total	203	359.8 ± 62.3	466	352.3 ± 55.3	264	361.1 ± 56.3	1111	353.8 ± 61.4	0.49
Men	72	344.1 ± 73.5	136	346.4 ± 63.6	60	355.4 ± 60.6	261	338.7 ± 70.4	0.33
Women	131	368.4 ± 53.5	330	354.7 ± 51.3	204	362.8 ± 55.0	850	358.5 ± 57.7	0.50
Sleep Efficiency									
Total	203	81.1 ± 11.4	466	81.6 ± 9.6	264	82.7 ± 9.0	1111	82.2 ± 10.0	0.11
Men	72	75.7 ± 14.0	136	78.1 ± 11.2	60	79.3 ± 9.9	261	77.4 ± 11.2	0.60
Women	131	84.0 ± 8.4	330	83.1 ± 8.5	204	83.7 ± 8.5	850	83.7 ± 9.1	0.73

^(a)^ Linear regression model.

**Table 3:** Poor sleep quality and insomnia symptoms discriminated by the frequency of forest walking.

Frequency of Forest Walking	Total		Once a Month or More		Several Times a Year		Once a Year		Rarely		*p* ^(a)^
	*n*	(%)	*n*	(%)	*n*	(%)	*n*	(%)	*n*	(%)	
Total	2044		203		466		264		1111		
PSQI > 5.5	884	(43.2)	73	(36.0)	189	(40.6)	106	(40.2)	516	(46.4)	0.001
ISI ≥ 10	628	(30.7)	46	(22.7)	139	(29.8)	76	(28.8)	367	(33.0)	0.005
Men	529		72		136		60		261		
PSQI > 5.5	222	(42.0)	22	(30.6)	56	(41.2)	18	(30.0)	126	(48.3)	0.009
ISI ≥ 10	158	(29.9)	19	(26.4)	45	(33.1)	14	(23.3)	80	(30.7)	0.82
Women	1515		131		330		204		850		
PSQI > 5.5	662	(43.7)	51	(38.9)	133	(40.3)	88	(43.1)	390	(45.9)	0.04
ISI ≥ 10	470	(31.0)	27	(20.6)	94	(28.5)	62	(30.4)	287	(33.8)	0.002

PSQI: Pittsburgh Sleep Quality Index; ISI: Insomnia Severity Index; ^(a)^ Mantel–Haenszel test for trend.

**Table 4:** Adjusted odds ratio (aOR) of frequency of forest walking for the prevalences of poor sleep quality and insomnia symptoms.

	*n*	Poor Sleep Quality ^(a)^				Insomnia Symptoms ^(b)^			
		Model 1 ^(e)^		Model 2 ^(f)^		Model 1 ^(e)^		Model 2 ^(f)^	
		aOR ^(c)^	95% CI ^(d)^	aOR	95% CI	aOR	95% CI	aOR	95% CI
Total									
Frequency of forest walking		*p* = 0.02		*p* = 0.04		*p* = 0.02		*p* = 0.03	
Once a month or more	203	1	Ref.	1	Ref.	1	Ref.	1	Ref.
Several times a year	466	1.19	(0.84–1.68)	1.17	(0.82–1.65)	1.53	(1.04–2.25)	1.52	(1.03–2.24)
Once a year	264	1.17	(0.79–1.71)	1.14	(0.77–1.67)	1.48	(0.96–2.28)	1.46	(0.95–2.25)
Rarely	1111	1.49	(1.18–2.04)	1.45	(1.05–2.00)	1.75	(1.22–2.51)	1.73	(1.20–2.48)
Men									
Frequency of forest walking		*p* = 0.03		*p* = 0.02		*p* = 0.47		*p* = 0.47	
Once a month or more	72	1	Ref.	1	Ref.	1	Ref.	1	Ref.
Several times a year	136	1.28	(0.68–2.40)	1.27	(0.67–2.38)	1.29	(0.67–2.48)	1.29	(0.67–2.48)
Once a year	60	0.73	(0.34–1.58)	0.74	(0.34–1.61)	0.73	(0.32–1.67)	0.73	(0.32–1.66)
Rarely	261	1.68	(0.95–3.00)	1.74	(0.98–3.11)	1.11	(0.60–2.05)	1.11	(0.60–2.05)
Women									
Frequency of forest walking		*p* = 0.20		*p* = 0.34		*p* = 0.01		*p* = 0.02	
Once a month or more	131	1	Ref.	1	Ref.	1	Ref.	1	Ref.
Several times a year	330	1.09	(0.72–1.66)	1.06	(0.70–1.62)	1.67	(1.02–2.74)	1.66	(1.01–2.72)
Once a year	204	1.27	(0.80–1.99)	1.21	(0.76–1.91)	1.93	(1.14–3.27)	1.86	(1.09–3.16)
Rarely	850	1.37	(0.93–2.02)	1.30	(0.88–1.92)	2.11	(1.34–3.34)	2.04	(1.29–3.23)

^(a)^ Pittsburgh Sleep Quality Index > 5.5; ^(b)^ Insomnia Severity Index ≥ 10; ^(c)^ adjusted odds ratio; ^(d)^ confidence interval; ^(e)^ adjustment by age (39–49, 50s, 60s, and 70s), body mass index (≥25.0/<25.0), smoking status (current smoker/other responses), habitual nightcap use (once a week or more/other responses), and habitual exercise (leisure time activity: once a week for at least 30 min or more/other responses); ^(f)^ adjusted by age, body mass index, smoking status, habitual nightcap use, habitual exercise, and chronotype (morning type, intermediate type, and evening type).

## Data Availability

Data are contained within the article.
